# Assessment of carbapenems in a mouse model of *Mycobacterium tuberculosis* infection

**DOI:** 10.1371/journal.pone.0249841

**Published:** 2021-05-03

**Authors:** Ravindra Jadhav, Ricardo Gallardo-Macias, Gaurav Kumar, Samer S. Daher, Amit Kaushik, Kristina M. Bigelow, Eric L. Nuermberger, Gyanu Lamichhane, Joel S. Freundlich

**Affiliations:** 1 Department of Pharmacology, Physiology, and Neuroscience, Rutgers University–New Jersey Medical School, Newark, New Jersey, United States of America; 2 Center for Tuberculosis Research and Department of Medicine, Johns Hopkins University, Baltimore, MD, United States of America; 3 Division of Infectious Disease, Department of Medicine and the Ruy V. Lourenço Center for the Study of Emerging and Re-emerging Pathogens, Rutgers University—New Jersey Medical School, Newark, New Jersey, United States of America; Newcastle University, UK, UNITED KINGDOM

## Abstract

We present further study of a subset of carbapenems, arising from a previously reported machine learning approach, with regard to their mouse pharmacokinetic profiling and subsequent study in a mouse model of sub-acute *Mycobacterium tuberculosis* infection. Pharmacokinetic metrics for such small molecules were compared to those for meropenem and biapenem, resulting in the selection of two carbapenems to be assessed for their ability to reduce *M*. *tuberculosis* bacterial loads in the lungs of infected mice. The original syntheses of these two carbapenems were optimized to provide multigram quantities of each compound. One of the two experimental carbapenems, JSF-2204, exhibited efficacy equivalent to that of meropenem, while both were inferior to rifampin. The lessons learned in this study point toward the need to further enhance the pharmacokinetic profiles of experimental carbapenems to positively impact *in vivo* efficacy performance.

## Introduction

Tuberculosis represents a global health pandemic caused by *Mycobacterium tuberculosis* infection. It is characterized by approximately ten million new cases and 1.4 million deaths per year [1]. Multi-drug resistant (MDR) cases, defined by resistance to at least isoniazid and rifampin, constituted nearly one-half million cases in 2019 [1]. Although the recent regulatory approvals of bedaquiline and pretomanid have introduced two new tuberculosis drugs, reports of resistance emerging to either drug reinforce the urgency of developing additional new agents to enable regimens comprised entirely of new agents [2,3].

While β-lactams are arguably the most successful class of antibacterials and the carbapenem sub-class, in particular, occupies a significant niche in treating life-threatening, drug-resistant infections, this family of molecules is currently absent from front-line tuberculosis treatments [4]. However, interest in using carbapenems to treat MDR-TB has increased significantly, as evidenced by numerous case reports, case series, and reviews describing their use [5–12]. More important to understanding the potential contribution of this class, a phase 2 study (NCT02349841) recently demonstrated substantial early bactericidal activity of a globally marketed injectable carbapenem, meropenem [8] (co-administered with amoxicillin/clavulanate to inhibit the *M*. *tuberculosis* β-lactamase, BlaC). The results showed a promising reduction of bacterial load in patients’ sputum as compared to standard four-drug therapy [9]. However, the clinical utility of meropenem remains limited due to the fact that it must be administered as an intravenous infusion every 8 h, which is not conducive to widespread use in the field. In addition, it must be administered with clavulanate for optimal activity. Faropenem is an efficacious inhibitor of *M*. *tuberculosis* growth *in vitro* [13,14], but still may not be potent enough to reach effective concentrations when delivered orally as the sodium salt (even thrice daily) at doses used for other indications [15,16]. Therefore, the availability of more potent carbapenems with improved pharmacokinetics and oral bioavailability clearly will remain an unmet need in combating MDR-TB [14,17]. Our previous publication in this area outlined the use of Bayesian modeling and structure-based design tenets, to design a new set of carbapenems and profile their biological activity [6]. Herein, we report our continued studies to pursue these carbapenems, with a specific focus on evaluating the pharmacokinetic (PK) profile and efficacy of select compounds against *M*. *tuberculosis* in mice.

## Materials and methods

### Synthetic chemistry procedures and spectroscopic data

All reagents were purchased from commercial suppliers and used without further purification unless noted otherwise. All chemical reactions occurring solely in an organic solvent were carried out under an inert atmosphere of argon or nitrogen. Analytical TLC was performed with Merck silica gel 60 F_254_ plates. Column chromatography was conducted with Teledyne Isco CombiFlash Companion or Rf+ systems. ^1^H NMR spectra were acquired on Varian Inova 400, 500, and 600 MHz instruments and are listed in parts per million downfield from TMS. LC-MS was performed on an Agilent 1260 HPLC coupled to an Agilent 6120 MS. All synthesized compounds were at least 95% pure as judged by their HPLC trace at 250 nm and were characterized by the expected parent ion(s) in the MS trace.

### Synthesis of 4-nitrobenzyl (4*R*,5*S*,6*S*)-6-((*R*)-1-hydroxyethyl)-4-methyl-7-oxo-3-(phenethylthio)-1-azabicyclo[3.2.0]hept-2-ene-2-carboxylate (compound 2)

A solution of 4-nitrobenzyl (4*R*,5*S*,6*S*)-3-[(diphenylphosphono)oxy]-6-[(*R*)-1-hydroxyethyl]-4-methyl-7-oxo-1-azabicyclo[3.2.0]hept-2-ene-2-carboxylate (compound **1**; 15.0 g, 25.2 mmol) in dimethylformamide (90 mL) was cooled to 0°C, and N,N-di-*i*-propylethylamine (9.30 mL, 50.4 mol) followed by 2-phenylethane-1-thiol (4.80 g, 30.2 mmol) were added. The reaction mixture was stirred for 1 h at 0°C. EtOAc (360 mL) was then added, and the organic layer was washed with saturated aqueous brine solution (3 x 100 mL). The organic layers were dried over anhydrous sodium sulfate, filtered, and concentrated *in vacuo*. The crude product was purified by flash chromatography using hexane/EtOAc (6:4) to afford the desired product as an off-white solid (10.3 g, 21.3 mmol, 84.5%): ^1^H NMR (500 MHz, CDCl_3_) δ 8.21 (d, *J* = 8.5 Hz, 2), 7.65 (d, *J* = 8.5 Hz, 2), 7.32 (t, *J* = 7.4 Hz, 2), 7.26 (dd, *J* = 13.9, 6.9 Hz, 1), 7.21 (d, *J* = 7.4 Hz, 2), 5.51 (d, *J* = 13.8 Hz, 1), 5.24 (d, *J* = 13.8 Hz, 1), 4.25 (p, *J* = 6.3 Hz, 1), 4.14 (dd, *J* = 9.1, 2.2 Hz, 1), 3.36–3.28 (m, 1), 3.26 (dd, *J* = 6.7, 2.2 Hz, 1), 3.16–3.02 (m, 2), 3.02–2.89 (m, 2), 1.35 (d, *J* = 6.2 Hz, 3), 1.23 (t, *J* = 9.3 Hz, 3). One H was unaccounted for and presumably was the OH; ^13^C NMR (126 MHz, CDCl_3_) δ 172.8, 160.5, 152.9, 147.6, 143.2, 139.2, 128.8, 128.6, 128.2, 126.9, 123.8, 123.4, 65.9, 65.3, 59.6, 56.1, 43.3, 36.3, 33.0, 21.9, 16.9. LRMS (ESI): Calculated for C_25_H_27_N_2_O_6_S (M+H)^+^ = 483.2; Observed 483.2.

### Sodium (4*R*,5*S*,6*S*)-6-((*R*)-1-hydroxyethyl)-4-methyl-7-oxo-3-(phenethylthio)-1-azabicyclo[3.2.0]hept-2-ene-2-carboxylate (JSF-2417)

To a solution of 4-nitrobenzyl (4*R*,5*S*,6*S*)-6-((*R*)-1-hydroxyethyl)-4-methyl-7-oxo-3-(phenethylthio)-1-azabicyclo[3.2.0]hept-2-ene-2-carboxylate (compound **2**; 8.00 g, 16.6 mol) in a mixture of *n*-butanol (50 mL) and water (50 mL) was added sodium bicarbonate (1.53 g, 18.2 mol). The reaction mixture was subjected to catalytic hydrogenation at 60 psi pressure in a Parr shaker hydrogenation apparatus in the presence of 10% (w/w) palladium on charcoal (4.00 g) for 1 h. After completion of the reaction, the reaction mixture was filtered through a pad of Celite and washed with water (10 mL). The aqueous layer was washed with diethyl ether (25 x 3 mL) and concentrated under reduced pressure. The residue was purified by chromatography on a reverse-phase C18 column using a solvent system of water/acetonitrile to provide the desired compound as a white solid (4.28 g, 11.6 mol, 69.8%): ^1^H NMR (500 MHz, D_2_O) δ 7.34 (t, *J* = 7.2 Hz, 2), 7.28 (t, *J* = 8.9 Hz, 3), 4.19 (p, *J* = 6.0 Hz, 1), 3.88 (d, *J* = 9.0 Hz, 1), 3.29 (d, *J* = 5.8 Hz, 1), 3.14–3.01 (m, 2), 2.98–2.82 (m, 3), 1.27 (d, *J* = 6.3 Hz, 3), 1.05 (d, *J* = 7.1 Hz, 3); Two hydrogens were unaccounted for and were presumably the OHs. ^13^C NMR (126 MHz, D_2_O) δ 176.2, 167.9, 143.6, 139.9, 130.1, 129.1, 128.6, 126.7, 65.1, 58.0, 55.7, 41.9, 35.99, 32.0, 20.2, 15.9. Calculated for C_18_H_22_NO_4_S (M+H)^+^ = 348.1; Observed 348.0.

### 4-nitrobenzyl (4*R*,5*S*,6*S*)-6-((*R*)-1-hydroxyethyl)-4-methyl-3-(methylthio)-7-oxo-1-azabicyclo[3.2.0]hept-2-ene-2-carboxylate (compound 3)

To a 500 mL round-bottom flask, 200 mL dimethylformamide was charged, cooled to 0°C, and 3M sodium methanethiolate (4.20 g, 59.9 mmol), 1,1,3,3-tetramethylguanidine (10.6 mL, 89.7 mmol), and acetic acid (4.3 mL, 72 mmol) were added. The reaction mixture was stirred for 5 min at 0°C, was then cooled to -40°C, and a solution of 4-nitrobenzyl (4*R*,5*S*,6*S*)-3-[(diphenylphosphono)oxy]-6-[(*R*)-1-hydroxyethyl]-4-methyl-7-oxo-1-azabicyclo[3.2.0]hept-2-ene-2-carboxylate (32.0 g, 53.9 mmol) in 100 mL dimethylformamide was added. After stirring at -40°C for 90 min, the reaction mixture was diluted with 500 mL ethyl acetate and washed with water (3 x 250 mL). The organics were washed with saturated aqueous brine solution (1 x 100 mL), dried over anhydrous sodium sulfate, and concentrated *in vacuo*. The crude product was purified by flash chromatography using hexane/EtOAc (6:4) to afford the desired product as an off-white solid (10.8 g, 27.5 mmol, 51.0%): ^1^H NMR (500 MHz, CDCl_3_) δ 8.21 (d, *J* = 8.1 Hz, 2), 7.65 (d, *J* = 8.2 Hz, 2), 5.50 (d, *J* = 13.8 Hz, 1), 5.24 (d, *J* = 13.8 Hz, 1), 4.30–4.20 (m, 2), 3.43 (p, *J* = 7.6 Hz, 1), 3.27 (d, *J* = 6.9 Hz, 1), 2.41 (s, 3), 1.78 (br s, 1), 1.37 (d, *J* = 6.2 Hz, 3), 1.27 (d, *J* = 7.2 Hz, 3). Calculated for C_18_H_21_N_2_O_6_S (M+H)^+^ = 393.1; Observed 393.0.

### Sodium (4*R*,5*S*,6*S*)-6-((*R*)-1-hydroxyethyl)-4-methyl-3-(methylthio)-7-oxo-1-azabicyclo[3.2.0]hept-2-ene-2-carboxylate (JSF-2204)

To a solution of 4-nitrobenzyl (4*R*,5*S*,6*S*)-6-((*R*)-1-hydroxyethyl)-4-methyl-3-(methylthio)-7-oxo-1-azabicyclo[3.2.0]hept-2-ene-2-carboxylate (compound **3**; 6.48 g, 16.5 mmol) in a mixture of *n*-butanol (50 mL) and water (50 mL) was added sodium bicarbonate (1.52 g, 18.1 mmol). The reaction mixture was subjected to catalytic hydrogenation at 60 psi pressure in a Parr shaker hydrogenation apparatus in the presence of 10% (w/w) palladium on charcoal (3.25 g) for 1 h. After completion of the reaction, the reaction mixture was filtered through a pad of Celite and washed with water (10 mL). The aqueous layer was washed with diethyl ether (25 x 3 mL) and concentrated *in vacuo*. The residue was purified by chromatography on a reverse-phase C_18_ column using a solvent system of water/acetonitrile. The material was lyophilized to afford the desired product (2.40 g, 8.60 mmol, 52.1%) as a white solid: ^1^H NMR (500 MHz, D_2_O) δ 4.20–4.11 (m, 1), 4.07 (dd, *J* = 8.8, 2.0 Hz, 1), 3.45–3.35 (m, 1), 3.30 (dd, *J* = 6.3, 2.0 Hz, 1), 2.28 (s, 3), 1.21 (t, *J* = 9.9 Hz, 3), 1.11 (d, *J* = 7.2 Hz, 3); Two hydrogens were unaccounted for and were presumably the OHs. ^13^C NMR (126 MHz, D_2_O) δ 176.0, 168.3, 146.8, 128.1, 65.3, 57.8, 56.0, 41.7, 20.1, 15.9, 13.7. Calculated for C_11_H_16_NO_4_S (M+H)^+^ = 258.1; Observed 258.0.

### Sodium (4*R*,5*S*,6*S*)-6-((*R*)-1-hydroxyethyl)-4-methyl-3-((3-morpholino-3-oxopropyl)thio)-7-oxo-1-azabicyclo[3.2.0]hept-2-ene-2-carboxylate (JSF-2700)

A solution of 4-nitrobenzyl (4*R*,5*S*,6*S*)-3-[(diphenylphosphono)oxy]-6-[(*R*)-1-hydroxyethyl]- 4-methyl-7-oxo-1-azabicyclo[3.2.0]hept-2-ene-2-carboxylate (0.66 g, 1.1 mmol) in dimethylformamide (3 mL) was cooled to 0°C, and N,N-di-*i*-propylethylamine (0.24 mL, 1.37 mmol) followed by 3-mercapto-1-morpholinopropan-1-one (0.240 g, 1.37 mmol) were added. The reaction mixture was stirred for 12 h at 0°C and was then warmed gradually to rt. EtOAc (5 mL) was then added, and the organic layer was washed with saturated aqueous brine solution (3 x 5 mL). The organics were dried over anhydrous sodium sulfate, filtered, and concentrated *in vacuo*. The crude product was purified by flash chromatography using CH_2_Cl_2_/MeOH (9:1) to afford the desired product 4-nitrobenzyl (4*R*,5*S*,6*S*)-6-((*R*)-1-hydroxyethyl)-4-methyl-3-((3-morpholino-3-oxopropyl)thio)-7-oxo-1-azabicyclo[3.2.0]hept-2-ene-2-carboxylate as a white solid (555 mg, 1.07 mmol, 97.1%): ^1^H NMR (500 MHz, CDCl_3_) δ 8.22 (d, *J* = 8.7 Hz, 2), 7.65 (d, *J* = 8.7 Hz, 2), 5.50 (d, *J* = 13.8 Hz, 1), 5.23 (d, *J* = 13.8 Hz, 1), 4.39–4.15 (m, 2), 3.65 (s, 4), 3.61 (dd, *J* = 9.5, 4.5 Hz, 2), 3.52 (dt, *J* = 14.7, 7.3 Hz, 1), 3.46–3.41 (m, 2), 3.28 (dd, *J* = 6.6, 2.5 Hz, 1), 3.22 (ddd, *J* = 12.9, 8.4, 6.5 Hz, 1), 3.14 (ddd, *J* = 12.8, 8.6, 6.4 Hz, 1), 2.77–2.52 (m, 2), 1.36 (d, *J* = 6.3 Hz, 3), 1.28 (d, *J* = 7.3 Hz, 3). One H was unaccounted for and presumably was an OH. Calculated for C_24_H_30_N_3_O_8_S (M+H)^+^ = 520.2; Observed 520.0.

To a solution of 4-nitrobenzyl (4*R*,5*S*,6*S*)-6-((*R*)-1-hydroxyethyl)-4-methyl-3-((3-morpholino-3-oxopropyl)thio)-7-oxo-1-azabicyclo[3.2.0]hept-2-ene-2-carboxylate (0.034 g, 0.065 mmol) in a mixture of ethanol (2 mL), water (2 mL), and EtOAc (1 mL) was added sodium bicarbonate (0.010 g, 0.12 mmol). The reaction mixture was subjected to catalytic hydrogenation at 60 psi pressure in the presence of 10 percent (w/w) palladium on charcoal (2 mg) for 1 h. After completion of the reaction, the reaction mixture was filtered through a pad of Celite and washed with water (5 mL). The aqueous layer was washed with diethyl ether (7 x 3 mL) and concentrated *in vacuo*. The residue was purified by chromatography on a reverse-phase C_18_ column using a solvent system of water/acetonitrile to provide the desired compound as an off-white solid (22 mg, 0.054 mmol, 81%): ^1^H NMR (500 MHz, D_2_O) δ 4.25 (s, 1), 4.18 (d, *J* = 9.0 Hz, 1), 3.73 (s, 4), 3.60 (dd, *J* = 28.9, 8.6 Hz, 4), 3.46 (dd, *J* = 15.5, 8.0 Hz, 1), 3.23–3.14 (m, 1), 3.04–2.95 (m, 1), 2.79 (s, 2), 1.30 (d, *J* = 5.5 Hz, 3), 1.20 (d, *J* = 6.6 Hz, 3). One H was unaccounted for and presumably was an OH; ^13^C NMR (126 MHz, D_2_O) δ 170.4, 148.2, 146.8, 127.6, 123.8, 66.3, 62.9, 60.5, 57.9, 50.7, 45.9, 32.4, 26.8 24.2, 20.6, 15. 1, 13.2. Calculated for C_17_H_25_N_2_O_6_S (M+H)^+^ = 385.1; Observed 385.2.

### Biological assay protocols

#### Bacterial strains, growth conditions, and drug controls

*M*. *tuberculosis* reference strain H_37_Rv (ATCC 25618) was used in this study. Cultures of this strain were grown in Middlebrook 7H9 complete broth (Difco) supplemented with 0.5% glycerol, 10% oleic acid-albumin-dextrose-catalase (OADC) and 0.05% Tween-80 as described previously [18] at 37°C with constant shaking. Organ homogenates were inoculated and *M*. *tuberculosis* colony-forming units (CFUs) were grown in Middlebrook selective 7H11 agar plates supplemented with 0.5% glycerol, 10% oleic acid-albumin-dextrose-catalase (OADC) and 0.05% Tween-80 at 37°C. Rifampin, meropenem, and clavulanate were obtained from Sigma-Aldrich.

#### Determination of minimum inhibitory concentration (MIC) against *M*. *tuberculosis*

For determination of the MIC of drugs against *M*. *tuberculosis* H_37_Rv, the standard micro broth dilution method was used as previously described [19]. Briefly, 10^5^ *M*. *tuberculosis* bacilli from exponentially growing culture were inoculated into wells of a 96-well microtiter dish with Middlebrook 7H9 broth without Tween-80 containing a drug at two-fold dilution ranging from 64 – 0.125 μg/mL. β-lactamase inhibitor, such as clavulanate, was not included in the MIC determinations and, therefore, the MIC values reported here were for the drugs alone. The final volume of the bacterial suspension with drug was 150 μL in a 350 μL volume well. Culture broth without drug alone was included as a negative control and with 10^5^ *M*. *tuberculosis* bacilli as a positive control. Cultures were incubated at 37°C in a standing incubator and growth or lack thereof was assessed after 14 d via visual inspection of the size of the bacterial pellet. Sizes of *M*. *tuberculosis* pellet at the bottom of the U-shaped wells were recorded. The lowest concentration at which we were unable to observe any pellet were reported as the drug MIC.

#### Evaluation of drug pharmacokinetics and efficacy in mice

All studies in mice were undertaken in accordance with a protocol approved by the Johns Hopkins Animal Care and Use Committee (MO17M279). For determination of level of a compound in plasma, 5–6 week old, female BALB/c mice (Jackson Laboratory) were used. Five experimental carbapenems were evaluated and two commercially available carbapenems, meropenem and biapenem, were included as controls. A single dose of 25 mg/kg was injected subcutaneously into the dorsal flank of the hind limb using a 100 μL bolus per mouse, three mice per compound. At 0.25, 0.50, 1, and 3 h following compound administration, blood was collected from tail vein into heparin vials and plasma was separated via centrifugation at 2,000 xg for 5 min. The concentration of each compound in the plasma samples was determined using a commercially available service with BioDuro (BioDuro, San Diego) relying on LC-MS quantification. A sub-acute mouse model of *M*. *tuberculosis* H_37_Rv was used to evaluate the efficacies of experimental carbapenems. Female 5–6 weeks old BALB/c mice (Jackson Laboratory) were used. An exponentially growing culture of *M*. *tuberculosis* H_37_Rv was used to prepare an inoculating suspension of 10 mL at an optical density (A_600nm_) of 0.1 by diluting in Middlebrook 7H9 broth and was aerosolized to infect mice. Sixty mice were infected at once in a Glas-Col aerosol chamber using the following cycle: 15 min of preheat, 30 min of nebulization of *M*. *tuberculosis* H_37_Rv suspension, 30 min of cloud decay and 15 min of heat and UV decontamination. Five mice were sacrificed 24 h after infection, lungs were resected, homogenized in sterile 1x PBS, and dilutions were inoculated onto complete Middlebrook 7H10 agar plates and incubated at 37°C for four weeks at which time *M*. *tuberculosis* CFUs were enumerated. Similarly, lung *M*. *tuberculosis* CFUs were enumerated from 5 mice at 2 weeks post infection to determine CFU lung burden at the time of treatment initiation. At this time, mice were randomly sorted into five groups, 10 per group. The first group, labeled ‘untreated’ was a control group that did not receive any treatment. The second group, a positive control for tuberculosis drug, received 10 mg/kg rifampin, once daily by oral gavage. The third group received twice daily dose of 400 mg/kg meropenem (a direct comparator drug of the carbapenem class) supplemented with 75 mg/kg β-lactamase inhibitor clavulanate by subcutaneous injection. Two experimental groups were comprised of mice that received twice daily dose of either the carbapenem JSF-2204 or JSF-2417, 400 mg/kg, also supplemented with 75 mg/kg β-lactamase inhibitor clavulanate by subcutaneous injection. Daily refers to 7 d a week. Five mice per group were sacrificed at one and three weeks following start of treatment and *M*. *tuberculosis* burden in the lungs of mice were determined as described above.

### Data analysis

One way ANOVA test was used to analyze data. Pairwise comparison of efficacies of different treatment based on CFU data was performed using a Wilcoxon rank sum test.

## Results and discussion

**Fig 1:**
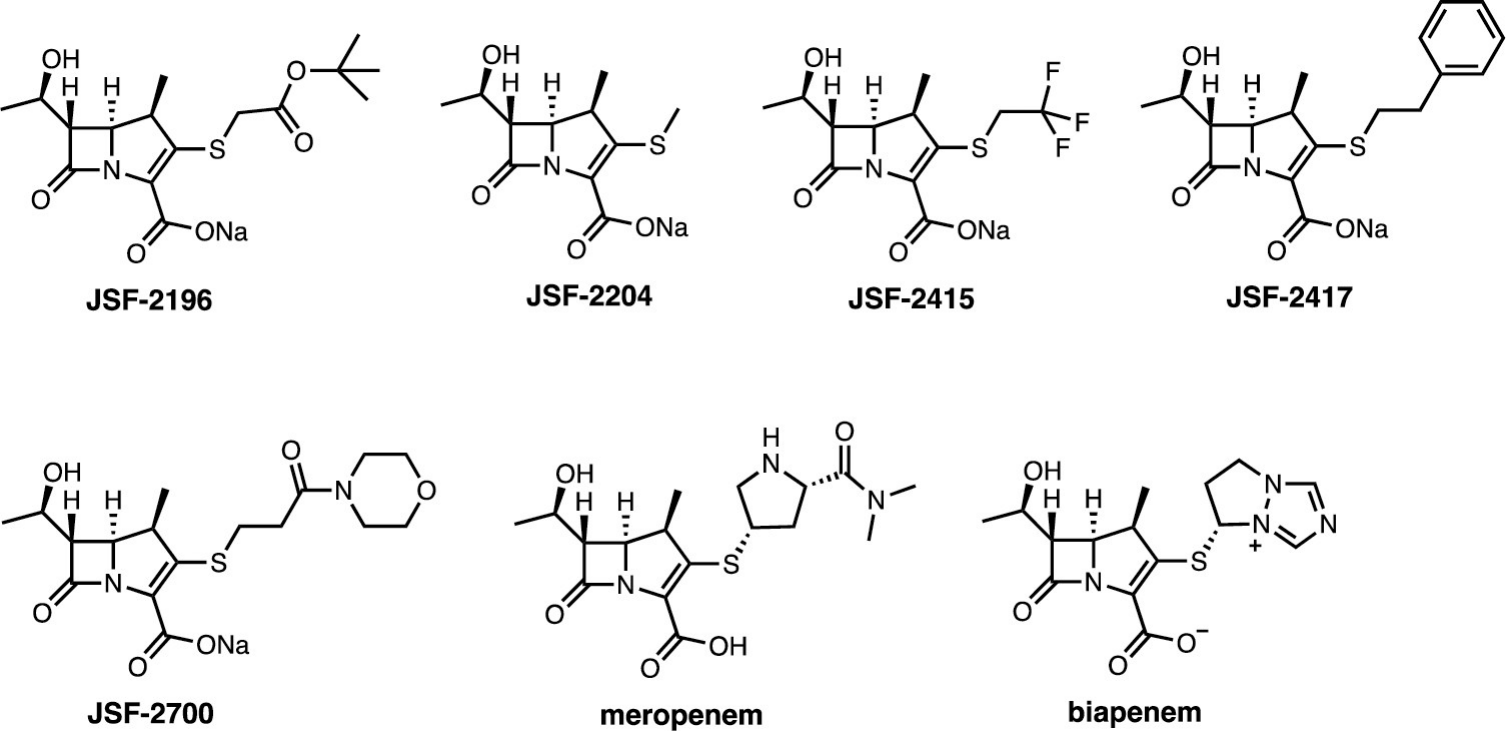
Chemical structures of the carbapenems studied.

**Fig 2:**
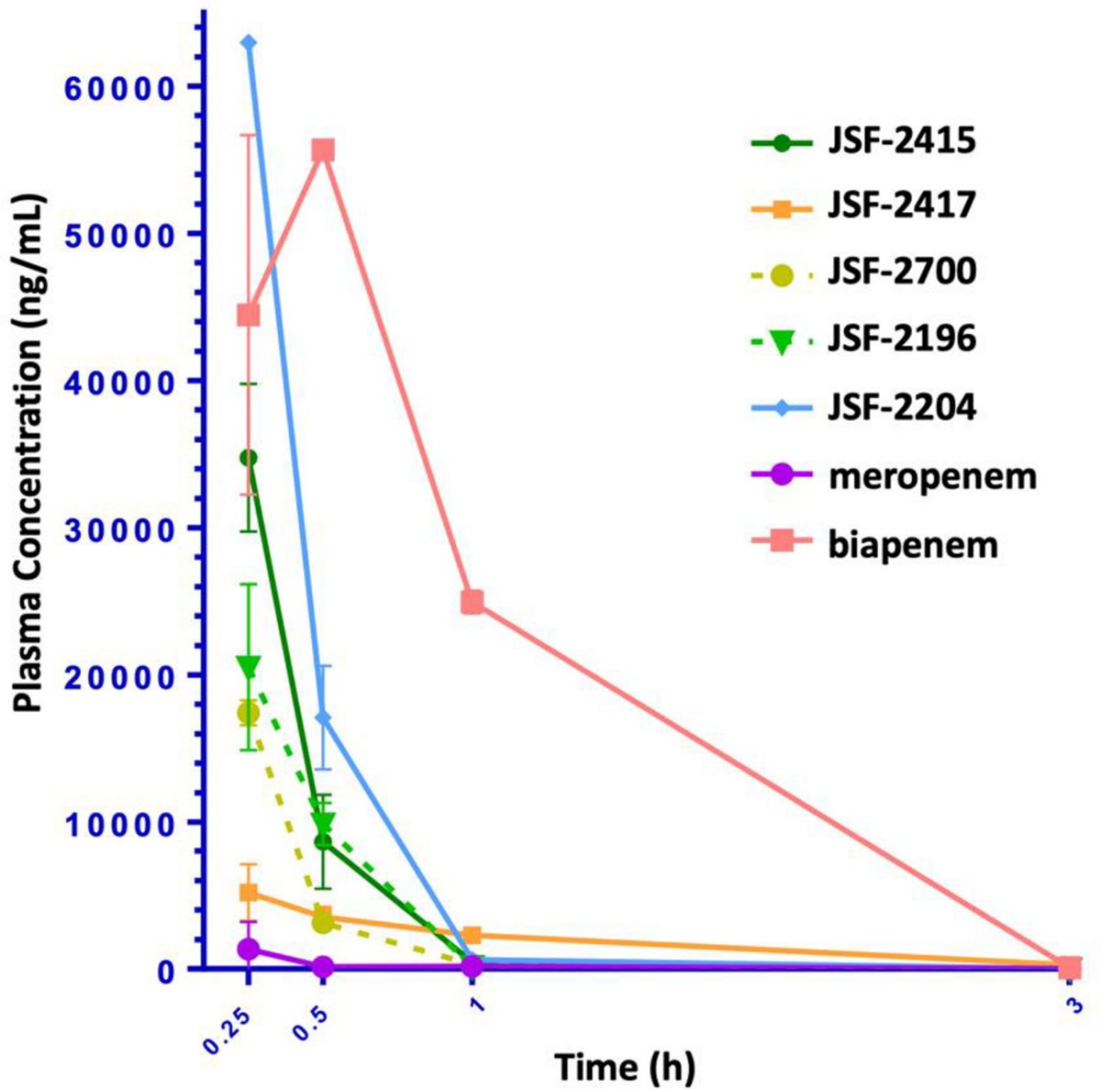
Plasma concentration versus time curves for the carbapenems post a single 25 mg/kg sc dose. Each data point represents the mean value and the error bars represent ± the standard deviation for 3 mice.

A subset of our previously reported carbapenems (JSF-2196, 2204, 2415, 2417) [6] and newly synthesized analog JSF-2700 (Fig 1), primarily chosen based on their minimum inhibitory concentration (MIC; the minimum concentration to inhibit growth by visual inspection of the bacterial pellet) values against *M*. *tuberculosis* H37Rv, were assayed for their PK profile in BALB/c mice with clinical carbapenems (meropenem, biapenem) included for comparison. A single subcutaneous (sc) injection of compound, formulated at 25 mg/kg in water, was administered to mice (group of three per compound) and the plasma concentration of compound was quantified over 3 h. A non-compartmental model was fit to the data (Fig 2 and Table 1). The systemic exposure was quantified as the area under the curve for the 3 h time period (AUC_0-3h_), the maximum plasma concentration (C_max_), the percentage of time above the MIC (%T>MIC), and the elimination half-life (t_1/2_). Analysis of the PK data for our carbapenems supported the *in vivo* efficacy study of JSF-2204. It exhibited the highest AUC_0-3h_ and C_max_ values while demonstrating a %T>MIC in the range of 33–100. Secondarily, JSF-2417 was also selected for further study. As compared to JSF-2415, JSF-2417 exhibited greater *in vitro* potency despite having lower values of AUC_0-3h_ and C_max_ values. JSF-2417 was also chosen over JSF-2700 as it exhibited a greater average plasma concentration at t = 3 h (261.5 ng/mL vs. 6.0 ng/mL).

**Table 1:** Summary of *in vitro* efficacy and mouse PK data for select carbapenems.

Cmpd	MIC (μg/mL)	t_1/2_ (h)	%T>MIC	C_max_ (ng/mL)	AUC_0-3h_ (h*ng/mL)
JSF-2196	2.0–4.0	0.230	17–33	20500	9056
JSF-2204	0.25–0.50	0.240	33–100	62950	22970
JSF-2415	4.0–8.0	0.241	17–33	34750	12547
JSF-2417	2.0–4.0	0.647	8.3–33	5190	5741
JSF-2700	2.0–4.0	0.258	8.3–33	17400	5933
Biapenem	2.5–5.0	0.289	33–100	55650	63264
Meropenem	4.0–8.0	0.463	<8.3	1336	613

**Fig 3:**
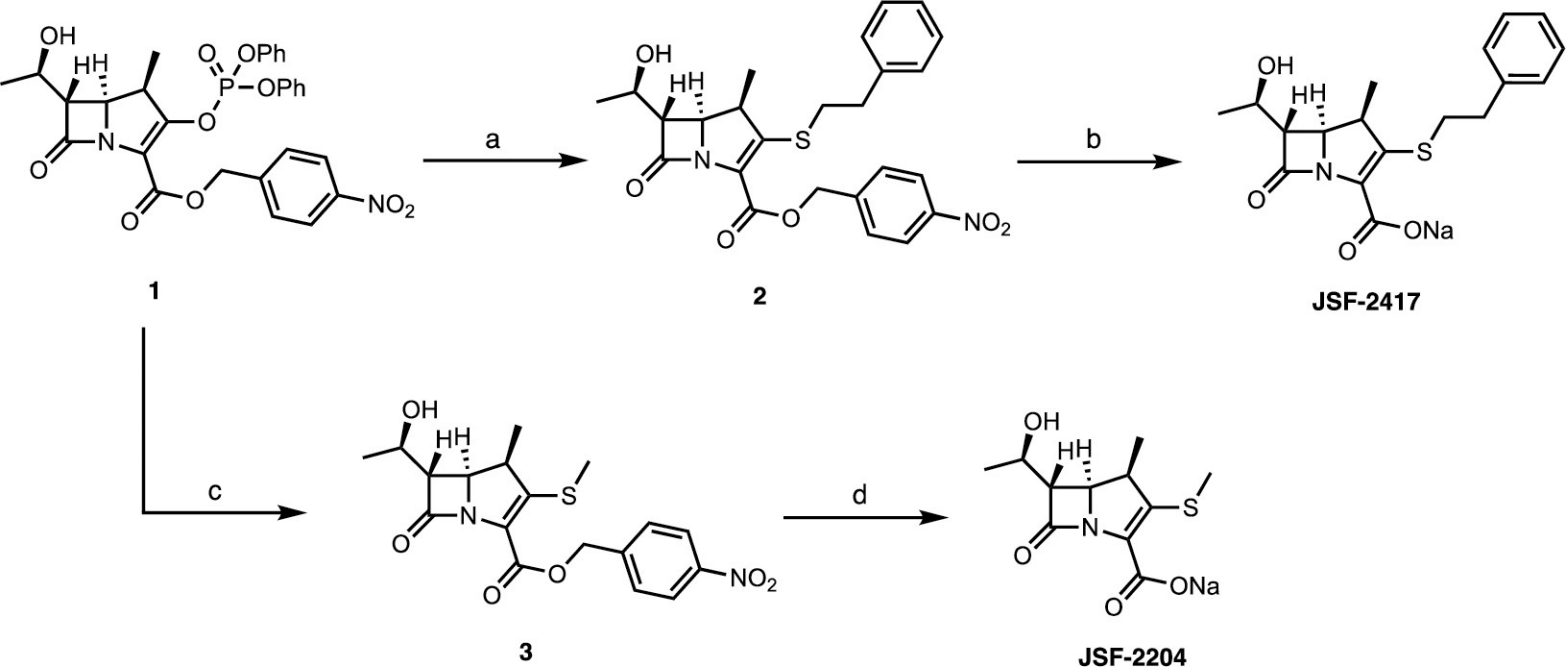
Scale-up routes for JSF-2417 and JSF-2204. Reagents and conditions: (a) PhCH_2_CH_2_SH, DIEA, DMF, 0°C, 1 h, 84%; (b) 60 psi H_2(g)_, 10% Pd/C, NaHCO_3_, *n-*BuOH/H_2_O (1:1), 1 h, 70%; (c) NaSMe, AcOH, TMG, DMF, -40°C, 50%; (d) 60 psi H_2(g)_, 10% Pd/C, NaHCO_3_, *n-*BuOH/H_2_O (1:1), 1 h, 52%.

We proceeded with the scale-up of the synthesis of JSF-2417 to facilitate *in vivo* efficacy studies. This was a significant challenge as our initial synthetic route to this compound was previously achieved on only a 100 mg scale. Synthesis of the protected carbapenem **2** was carried out by addition of 2-phenylethanethiol to the commercial vinyl phosphate **1** in the presence of N,N-di-*i*-propylethylamine (DIEA) with a yield of 84% on a 10 g scale (Fig 3). In contrast, our previously reported route [6] utilized di-*i-*propylamine in DMF and posed purification issues on scales beyond 250 mg. A range of conditions was then explored to achieve removal of the 4-nitrobenzyl moiety via hydrogenation with hydrogen gas in the presence of 10% w/w Pd/C (S1 Table). Conditions utilizing sodium bicarbonate in solvent mixtures such as EtOAc/EtOH/H_2_O afforded the desired product in low yields. A major impurity was observed due to β-lactam ring-opening as verified by LC-MS and ^1^H NMR. The hydrogenation reaction was next carried out in a variety of solvent systems in the absence of sodium bicarbonate. Unfortunately, in most of the cases, the reaction required more than 48 h to complete, again leading to β-lactam ring-opening. At 60 psi hydrogen pressure in solvents such EtOAc, EtOH, or THF, the reaction did not occur to any measurable extent. Ultimately, the catalytic hydrogenation was found to be successful when utilizing a 1:1 mixture of *n*-BuOH and water as the solvent system in conjunction with sodium bicarbonate to afford the desired compound in 70% yield on a >4 g scale. The scale-up of JSF-2204 began with the synthesis of methyl sulfide **3**. Our previous route involved generation of methanethiol, from sodium methyl sulfide and aqueous hydrochloric acid, and its subsequent reaction with phosphate **1** in the presence of DIEA with the overall yield of **3** being 52% on only a 400 mg scale [6]. We then explored other conditions that led to the use of sodium methyl sulfide, acetic acid, and 1,1,3,3-tetramethylguanidine (TMG) in DMF at -40°C. We prepared ca. 11 g of **3** in a 51% yield. Hydrogenation of the 4-nitrobenzyl ester in **3** under our previously reported [6] conditions (1.1 equiv NaHCO_3_, 1 atm H_2(g)_, THF/EtOH/H_2_O (1:1:1), 1 h) only afforded a 23% yield on a 4 g scale. Use of the hydrogenation conditions in the synthesis of JSF-2417 afforded JSF-2204 from intermediate **3** with a 52% yield on a 2 g scale.

**Fig 4:**
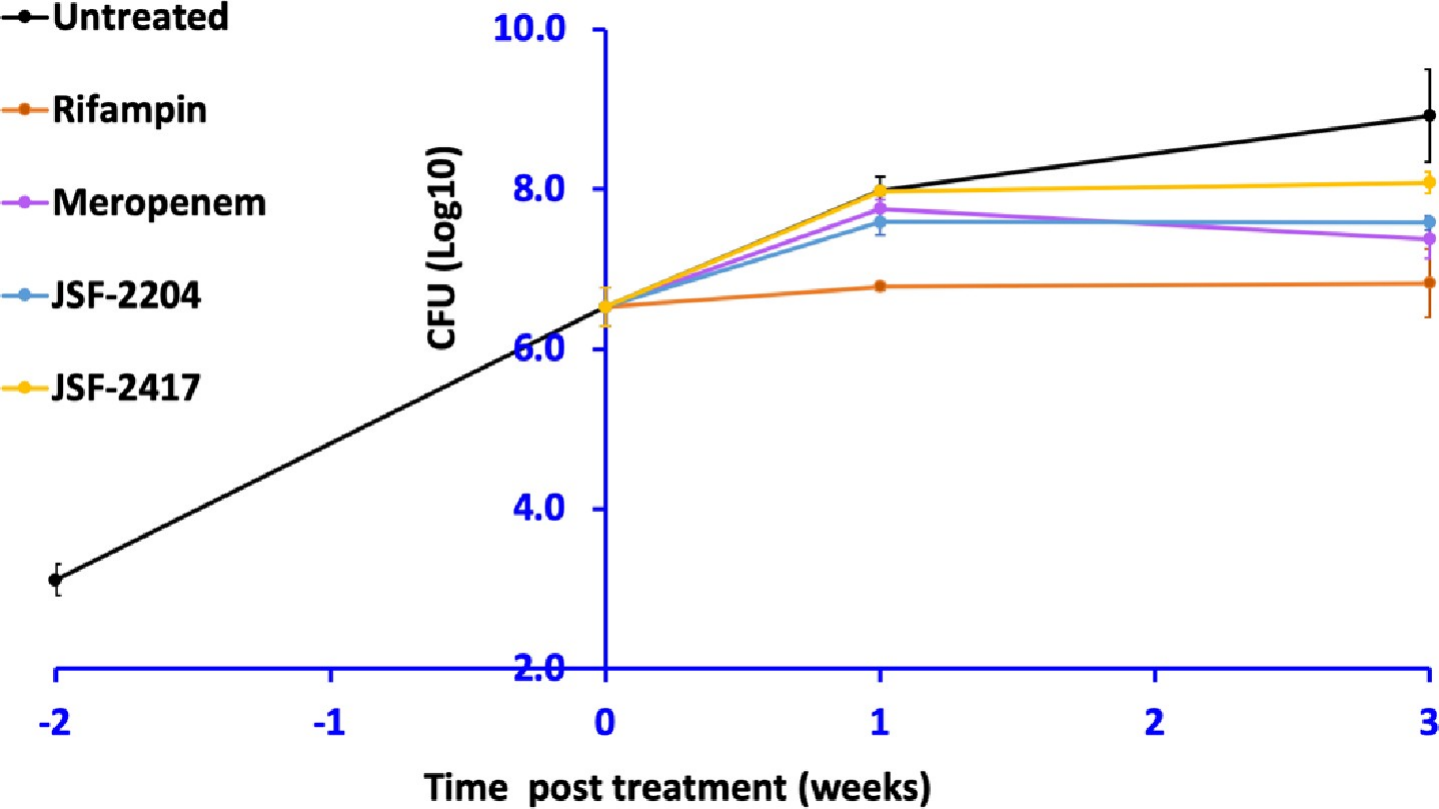
*M*. *tuberculosis* burden in the lungs of mice. Mean *M*. *tuberculosis* burdens in the lungs of five mice per group per time point, including standard deviation of the mean, are illustrated. Meropenem and experimental carbapenems JSF-2417 and JSF-2204 were administered sc at 400 mg/kg bid.

JSF-2417 and JSF-2204 were then evaluated in a mouse model of sub-acute *M*. *tuberculosis* infection. Female BALB/c mice were infected with the *M*. *tuberculosis* H37Rv strain as to afford a mean bacterial burden of ca. 10^6^ CFUs in the lungs two weeks post-infection. At that point, mice were treated with 400 mg/kg twice daily (bid) sc administration of JSF-2417, JSF-2204, or meropenem; rifampin served as a control with once daily administration orally at 10 mg/kg. Each carbapenem dose was supplemented with 75 mg/kg sodium clavulanate to inhibit the β-lactamase BlaC, in accord with previous studies [20]. The bacterial burden in mouse lungs was assessed in groups of five mice at 1 week and 3 weeks of treatment (Fig 4). After one week of dosing, the bacterial burden increased by ca. 1 log_10_ in the case of all three carbapenems; rifampin dosing led to a bacteriostatic effect. The bacteriostatic effect of rifampin was also observed after 3 weeks of dosing. Post 1 week of treatment, the bacterial load in the lungs failed to increase with continued dosing of each of the three carbapenems and ultimately each treatment prevented an increase in *M*. *tuberculosis* burden as compared to the untreated control. JSF-2204 exhibited similar efficacy as meropenem while JSF-2417 was inferior.

Based on superior %T>MIC, C_max_, and AUC_0-3h_ (Table 1) of JSF-2204 compared to meropenem, this new carbapenem can be expected to exhibit improved efficacy. However, JSF-2204 and meropenem exhibited statistically indistinguishable efficacy in the mouse model (Fig 4). It is possible that JSF-2204 is unable to penetrate the macrophages and other host cells harboring intracellular *M*. *tuberculosis* bacilli at the same rate as meropenem thereby failing to achieve sufficiently high local concentrations at these sites. The chemical modification present in JSF-2204 could potentially affect this attribute, eventually producing less than expected efficacy *in vivo*. The scope of the current study was to identify evolved carbapenems with improvement in *in vitro* activity and PK attributes over currently recommended carbapenem for treating drug-resistant tuberculosis, e.g., meropenem. JSF-2204 achieved this goal. In the future, we plan to improve the *in vivo* efficacy of JSF-2204 without sacrificing activity and PK advantages by generating analogs and testing them for *in vivo* efficacy.

Meropenem, JSF-52417, and JSF-2204 exhibited delayed activity in infected mice as little or no reduction in bacterial burden was observed in the lungs at the culmination of one week of treatment compared to the no-treatment group (Fig 4). Chambers et al. [7] and Kumar et al. [6] have also reported the delayed activity of imipenem and biapenem, respectively, in an acute *M*. *tuberculosis* infection model in mice.

The efforts disclosed herein demonstrate that structural modifications can be accommodated at the carbapenem 3-position to modulate PK and pharmacodynamic profiles. Efficacy evaluation of two modified carbapenems in *M*. *tuberculosis* infected mice afforded one compound, JSF-2204, that demonstrated statistically equivalent performance to meropenem. The *in vivo* activity observed for meropenem and our carbapenems to date must still be improved to afford single-agent efficacy data worthy of further translational progression. As in a study published by AstraZeneca, the efficacy shortcomings compared to current front-line drugs support further compound evolution to improve PK [14]. Future efforts will be required to design, synthesize, and biologically profile novel carbapenems to achieve *in vivo* efficacy superior to meropenem and on par with current antitubercular compounds of clinical relevance.

## Supporting information

S1 Table Hydrogenation conditions for removal of the 4-nitrobenzyl ester in intermediate 2. (DOC)
