# Supplementary material for: Assessment of carbapenems in a mouse model of Mycobacterium tuberculosis infection
Source: PLoS One. 2021 May 3;16(5):e0249841. doi: 10.1371/journal.pone.0249841 (PMC8092647; doi:10.1371/journal.pone.0249841)
Supplement: S1 Table — (DOC) [file pone.0249841.s001.doc]

*Supporting Information for*

**Assessment of carbapenems in a mouse model of *Mycobacterium tuberculosis* infection**

Ravindra Jadhav,^1,†^, Ricardo Gallardo-Macias^1,†^, Gaurav Kumar,^2^ Samer S. Daher^1^, Amit Kaushik,^2^ Eric L. Nuermberger,^2,*^ Gyanu Lamichhane,^2,*^ and Joel S. Freundlich^1,3,*^

1. Department of Pharmacology, Physiology, and Neuroscience, Rutgers University – New Jersey Medical School, Newark, New Jersey, 07103, USA.

2. Center for Tuberculosis Research and Department of Medicine, Johns Hopkins University, Baltimore, MD 21231

3. Division of Infectious Disease, Department of Medicine and the Ruy V. Lourenço Center for the Study of Emerging and Re-emerging Pathogens, Rutgers University - New Jersey Medical School, Newark, New Jersey 07103, USA.

**Table of Contents**

Table S1. Hydrogenation conditions for removal of the 4-nitrobenzyl ester in intermediate **2**

**Table S1.** Hydrogenation conditions for removal of the 4-nitrobenzyl ester in intermediate **2**.

| Trial | Reaction conditions | Substrate Scale (mg) | % Yield |
| --- | --- | --- | --- |
| 1 | 1 atm H_2_, 10% Pd/C, NaHCO_3_, EtOAc/EtOH/H_2_O (0.5:1:1) | 30 - 100 | 20-25 |
| 2 | 1 atm H_2_, 10% Pd/C, EtOAc/EtOH/H_2_O (0.5:1:1) | 100 | 0 |
| 3 | 1 atm H_2_, 10% Pd/C, EtOAc | 100 | 0 |
| 4 | 1 atm H_2_, 10% Pd/C, EtOH | 100 | 0 |
| 5 | 1 atm H_2_, 10% Pd/C, THF | 100 | 0 |
| 6 | 1 atm H_2_, 10% Pd/C, NMP (1.0 eq)/AcOH (1.0 eq), THF/H_2_O (1:1) | 600 | 60 |
| 7 | 1 atm H_2_, 10% Pd/C, EtOAc/H_2_O/NEP (1:2:1) | 100 | 55 |
| 8 | 1 atm H_2_, 10% Pd/C, DMF/H_2_O/NEP (1:1:0.7) | 100 | 50 |
| 9 | 1 atm H_2_, 10% Pd/C, dioxane/H_2_O/NEP (1:1:1) | 100 | 25 |
| 10 | 1 atm H_2_, 10% Pd/C, THF/H_2_O/NEP (1:1:0.3) | 500 | 35 |
| 11 | 1 atm H_2_, 10% Pd/C, *i-*amyl alcohol/H_2_O/NEP (1:1:1.5) | 100 | 15-20 |
| 12 | 1 atm H_2_, 10% Pd/C, *t-*BuOH/H_2_O/NEP (1:1:1.5) | 100 | 35 |
| 13 | 1 atm H_2_, 10% Pd/C, EtOAc/H_2_O/NEP (3:1:1), 2 d | 300 | 45 |
| 14 | 1 atm H_2_, 10% Pd/C, EtOAc/H_2_O/NEP (50:1:1), 2 d | 800 | 45-50 |
| 15 | 1 atm H_2_, 10% Pd/C, H_2_O/NEP (1:1), 2 d | 300 | 50-60 |
| 16 | 1 atm H_2_, 10% Pd/C, *n-*BuOH/H_2_O (1:1), 2 d | 300 | 30 |
| 17 | 60 psi H_2_, 10% Pd/C, EtOAc, 8 h | 300 | 0 |
| 18 | 60 psi H_2_, 10% Pd/C, EtOH, 8 h | 300 | 0 |
| 19 | 60 psi H_2_, 10% Pd/C, THF, 8 h | 300 | 0 |
| 20 | 60 psi H_2_, 10% Pd/C, NaHCO_3_, *n-*BuOH/H_2_O (1:1), 2 h | 1000 | 48 |
| 21 | 60 psi H_2_, 10% Pd/C, NaHCO_3_, *n-*BuOH/H_2_O (1:1), 2 h | 2000 | 58 |
| 22 | 60 psi H_2_, 10% Pd/C, NaHCO_3_, *n-*BuOH/H_2_O (1:1), 2 h | 5000 | 62 |
| 23 | 60 psi H_2_, 10% Pd/C, NaHCO_3_, *n-*BuOH/H_2_O (1:1), 2 h | 8000 | 70 |
